# Plant-Derived Anti-Inflammatory Compounds: Hopes and Disappointments regarding the Translation of Preclinical Knowledge into Clinical Progress

**DOI:** 10.1155/2014/146832

**Published:** 2014-05-29

**Authors:** Robert Fürst, Ilse Zündorf

**Affiliations:** Institute of Pharmaceutical Biology, Goethe-University Frankfurt, Max-von-Laue-Straße 9, 60438 Frankfurt/Main, Germany

## Abstract

Many diseases have been described to be associated with inflammatory processes. The currently available anti-inflammatory drug therapy is often not successful or causes intolerable side effects. Thus, new anti-inflammatory substances are still urgently needed. Plants were the first source of remedies in the history of mankind. Since their chemical characterization in the 19th century, herbal bioactive compounds have fueled drug development. Also, nowadays, new plant-derived agents continuously enrich our drug arsenal (e.g., vincristine, galantamine, and artemisinin). The number of new, pharmacologically active herbal ingredients, in particular that of anti-inflammatory compounds, rises continuously. The major obstacle in this field is the translation of preclinical knowledge into evidence-based clinical progress. Human trials of good quality are often missing or, when available, are frequently not suitable to really prove a therapeutical value. This minireview will summarize the current situation of 6 very prominent plant-derived anti-inflammatory compounds: curcumin, colchicine, resveratrol, capsaicin, epigallocatechin-3-gallate (EGCG), and quercetin. We will highlight their clinical potential and/or pinpoint an overestimation. Moreover, we will sum up the planned trials in order to provide insights into the inflammatory disorders that are hypothesized to be beneficially influenced by the compound.

## 1. Introduction

Inflammation is a crucial biological process for maintaining the body's homeostasis. It is indispensible for successfully fighting pathogens and for the repair of damaged tissue. However, inflammatory processes are also involved in the onset and maintenance of many severe disorders, such as rheumatoid arthritis, asthma, chronic inflammatory bowel diseases, type 2 diabetes, neurodegenerative diseases, and cancer [1]. The currently available repertoire of approved anti-inflammatory agents mainly consists of nonsteroidal anti-inflammatory drugs, glucocorticoids, immunosuppressant drugs, and biologicals. Despite this arsenal, therapy is often not effective enough or is hampered by intolerable side effects. Thus, the discovery of new anti-inflammatory compounds is still a great demand on scientists in academia and industry.

Plants were the first source of remedies in human history. In all cultures and through all ages different parts of a huge number of plants were used as drugs against all kinds of ailments. Needless to say, this usage was based on views that are not compatible with nowadays evidence-based medicine. However, different traditional systems, such as European-based plant collections, the Chinese Traditional Medicine, the Kampo system, the Indian Ayurveda, and many more, have evolved by trial and error and have strived toward advancing the appropriate use of plants on the background of their own, specific philosophy of life. One should be aware that in many lesser developed countries traditional medicine is still the only affordable and, thus, accessible way to meet the primary healthcare needs of a large number of patients.

The awareness that one (or more) chemical entity within the plant material is responsible for pharmacological actions and can be isolated for the use as single agent came up in the 19th century in the context of the emerging natural science-based medicine and pharmacy. Prominent plant-derived compounds that were isolated in this period, such as morphine, quinine, colchicine, atropine, pilocarpine, or theophylline, are still very important in current pharmacotherapy. Due to the developing organic synthesis, plant-derived compounds were among the first lead structures in the history of drug development.

Looking into the list of drugs approved within the last decades demonstrates that plant ingredients are still of importance in drug discovery. Vincristine, vinblastine, and their semisynthetic derivatives, originally isolated from the Madagascar periwinkle (*Catharanthus roseus*), are highly valuable anticancer drugs. Galantamine from the Caucasian snowdrop (*Galanthus caucasicus*), paclitaxel from the Pacific yew (*Taxus brevifolia*), and capsaicin from chili peppers (*Capsicum *species) are further prominent examples of secondary plant metabolites that made it into the clinic. Examples for plant-derived compounds that served as lead structures and/or were chemically modified are salicylic acid (acetylsalicylic acid), morphine (scores of derivatives), camptothecin (topotecan and irinotecan), artemisinin (artemether), and dicoumarol (warfarin).

The number of phytochemical studies describing new substances isolated from plants is huge and rising. Preclinical reports, that is,* in vitro*, cell-based, and animal studies on the action of these substances, are available in an inconceivable quantity. Unfortunately, these studies are often of equivocal quality, especially in the field of anti-inflammatory compounds. Also the review literature is overwhelming and there are many comprehensive publications available that focus on the respective molecular mechanisms [2–7].

Especially for newly isolated compounds, the knowledge is too often based on a very limited number of cell-based assays. From alterations of some prominent mediators of inflammatory processes, most frequently the transcription factor NF*κ*B, the compound is simply judged to be anti-inflammatory without presenting comprehensive* in vivo* data. Animal models are of course indispensible for the analysis of the pharmacological potential of a compound, but these models are used insufficiently and, as commonly known, do not satisfactorily reflect the situation in humans. The major problem is the lack of sound and significant clinical studies. In contrast, prominent and well-known plant-derived compounds, such as curcumin or resveratrol, have extensively been analyzed in clinical trials and have often induced a hype in media (even in scientific ones). However, due to reasons that will be discussed in the respective paragraphs, this knowledge has as yet not led to an approved drug that advances pharmacotherapy.

This minireview will summarize the current situation of 6 selected, prominent, anti-inflammatory compounds that have been tested in humans in recent years: curcumin, colchicine, resveratrol, capsaicin, epigallocatechin-3-gallate (EGCG), and quercetin. We will highlight the potential and/or pinpoint an overestimation of these agents. Moreover, we will sum up the planned registered trials in order to provide insights into the disorders that are hypothesized to be beneficially influenced by the compounds.

## 2. Plant-Derived Compounds Tested in Clinical Trials

### 2.1. Curcumin

**Figure 1:**
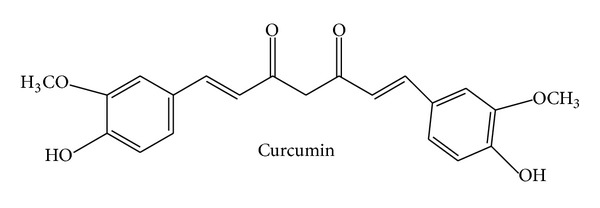


Curcumin (Figure 1), (1E,6E)-1,7-bis(4-hydroxy-3-methoxyphenyl)hepta-1,6-diene-3,5-dione, also known as diferuloylmethane, is the main ingredient of turmeric (*Curcuma longa*, Zingiberaceae). The Indian spice turmeric has been used for centuries in Ayurvedic medicine against inflammatory disorders. Curcumin was identified in 1910, is yellow in color, and represents a lipophilic polyphenol. It exerts a great variety of actions and is amongst the most frequently investigated natural compounds. Curcumin displays anti-inflammatory, antioxidant, anticancer/proapoptotic, and antibacterial activities due to a plethora of mechanisms. These have recently been reviewed comprehensively [8]. With regard to its anti-inflammatory action, curcumin was reported (i) to inhibit important proinflammatory signaling cascades, such as the NF*κ*B-, MAPK-, COX-, and LOX-pathways [9, 10], (ii) to downregulate the secretion of prominent cytokines, like TNF*α*, IL-1*β*, and IL-6 [11], and (iii) to block the expression of cell adhesion molecules (e.g., ICAM-1), which are necessary for the interaction of leukocytes with endothelial cells [12]. Thus, not only in regard to its anti-inflammatory profile, curcumin represents a compound with pleiotropic, multiple modes of action. Fortunately, the compound has been tested in humans. Currently, 77 studies (with known status) dealing with diverse actions of curcumin can be found on http://www.clinicaltrials.gov/. Thereof, 50 studies have been finished and 27 are in their onset (recruiting) phase. In PubMed, almost 100 clinical trials investigating curcumin in an inflammatory context are listed, of which 12 have been published in 2013, 24 in 2012, and 14 in 2011. These facts demonstrate that curcumin is still a hot topic and under intense clinical investigation. The most prominent disorders were rheumatoid arthritis, cancer (e.g., colorectal, pancreatic, breast, prostate, and lung), and inflammatory bowel diseases (ulcerative colitis and Crohn's disease), but there are also studies available, for example, on uveitis, vitiligo, or nephropathies, which reflects the pleiotropic actions of curcumin. A detailed list of these trials can be found in a recent review by Gupta et al. [13]. The recruiting future trials will mainly deal with the action of curcumin on cognitive impairment and—still ongoing—different types of cancer. Also inflammatory conditions will still be investigated. In these trials, curcumin often serves as a dietary supplement or as an adjunct treatment to the standard therapy. The Cochrane Collaboration lists one systematic review (published in 2012) that analyzes the effects of curcumin in ulcerative colitis [12, 14]. The authors conclude that “curcumin may be a safe and effective therapy for maintenance of remission in quiescent ulcerative colitis when given as adjunct therapy. However, further research in form of a large scale methodologically rigorous randomized controlled trial is needed” to really confirm a benefit. This statement perfectly reflects the overall situation. Clinical trials are available; however, they are often too weak and of poor quality to draw a clear conclusion. The major issue is the low number of enrolled patients, which frequently ranges between 10 and 30. It should also be mentioned that curcumin suffers from its very low bioavailability, although considerable progress has been made to overcome this obstacle by technological and chemical approaches [15]. Whether we will see curcumin as an approved (add-on) option for the treatment or prevention of one of the mentioned indications depends on the performance of solid, high-quality, big cohort studies in the future. However, from the wealth of studies, it can at least be concluded that curcumin seems to have a good safety profile; it is well tolerated and nontoxic.

### 2.2. Colchicine

**Figure 2:**
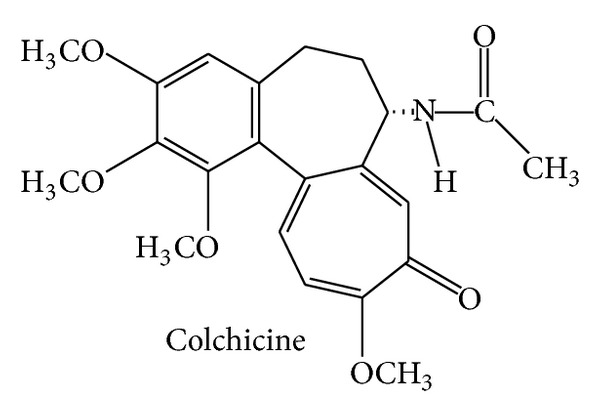


The tropolone derivative colchicine (Figure 2), N-[(7S)-1,2,3,10-tetramethoxy-9-oxo-6,7-dihydro-5H-benzo[a]heptalen-7-yl]acetamide, is the major alkaloid of the plant* Colchicum autumnale* (Colchicaceae), commonly known as autumn crocus or meadow saffron. Since ancient times, extracts of this plant have been used against gout attacks. Interestingly, the US FDA has only recently (2009) approved colchicine for the treatment of familial Mediterranean fever as well as for the treatment and prevention of acute gout flares. To get this approval, the applying company needed to provide new clinical data and, in return, was given an exclusive marketing agreement, 3 years for the indication gout and 7 years for familial Mediterranean fever (orphan drug status). The mechanisms of action of colchicine are well investigated: the molecular target was identified (tubulin), the binding site was precisely characterized, and the biological consequences of impairing microtubule dynamics were analyzed; comprehensive reviews summarizing these findings are available [16–19]. Noteworthy, colchicine played a crucial role for the initial characterization of microtubules and the tubulin subunits in the 1960s [20, 21]. Despite this huge knowledge and the fact that colchicine is already an approved drug, researchers recently have conducted several clinical studies in order to expand its fields of application. PubMed lists a number of very interesting trials in the field of inflammation-associated pathologies with positive outcome: colchicine was tested as adjunct treatment against acute [22, 23] and recurrent pericarditis [24, 25], for the prevention of atrial fibrillation after radiofrequency ablation [26] and for postpericardiotomy syndrome prevention [27]. These large and well-performed studies will surely affect pharmacotherapy guidelines. And the field is still vibrant: 14 clinical trials that are open, that is, in the recruiting phase, are listed on http://www.clinicaltrials.gov/. They intend to investigate the action of colchicine mainly in the areas of cardiology and nephrology, for example, in myocardial infarction, for postpericardiotomy syndrome prevention, or in diabetic nephropathy—all associated with inflammatory processes. Since the number of diseases with an inflammatory component is very large, one might speculate that colchicine will further stay an interesting, not yet fully exploited drug.

### 2.3. Resveratrol

**Figure 3:**
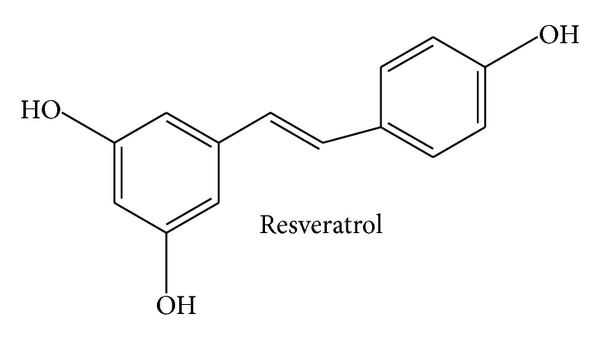


Resveratrol (Figure 3), 5-[(E)-2-(4-hydroxyphenyl)ethenyl]benzene-1,3-diol, represents a stilbene derivative and phytoalexin. It can be found in a great number of different plants and dietary products thereof, with peanuts, grapevines, and red wine being the most prominent ones. In parallel to curcumin, resveratrol was found to exert a wealth of pharmacological actions, for example, anti-inflammatory, antioxidant, anticancer/proapoptotic, chemopreventive, and antimicrobial properties. The number of available reviews on these topics is immense [28–37]. Resveratrol is known to have a poor bioavailability, which prompted many groups to work on improvement strategies [38–43]. As a consequence of these interesting* in vitro* and* in vivo* findings, a lot of effort was put into the elucidation of the underlying mechanisms of action. Again, a large number of reviews is available and gives in-depth insights into the mechanisms: resveratrol inhibits the NF*κ*B-, AP-1-, and COX-2-pathway [29, 44, 45] and activates PPAR, eNOS, and SIRT1 [46–49]. However, this gigantic knowledge has as yet not been translated into an approved clinical application. PubMed lists over 40 clinical trials on resveratrol in the broad context of inflammation-associated disorders, many of them dealing with diabetes, obesity, and coronary artery disease. These studies often analyze inflammation-related parameters in the plasma (e.g., CRP, TNF*α*, IL-1*β*, and IL-6) and in blood cells (e.g., activated kinases or transcription factors) or reported on functional parameters, such as the status of the endothelium [50–55]. Many trials convincingly demonstrate that these parameters are indeed beneficially influenced by resveratrol. However, whether this altered inflammatory status of the patients really results in a clinically relevant improvement of the severity of the diseases or, most importantly, in a reduced occurrence of disease-specific life-threatening events (not to mention mortality) has not been analyzed. According to http://www.clinicaltrials.gov/, 26 clinical trials on resveratrol are planned or are currently recruiting. The main field of interest is type 2 diabetes/metabolic syndrome, followed by polycystic ovary syndrome, nonalcoholic fatty liver disease, and mild cognitive impairment. Research would strongly profit by conducting interventional studies with defined primary outcomes reflecting the stage and/or prevalence of the diseases on a long-term basis.

### 2.4. Capsaicin

**Figure 4:**
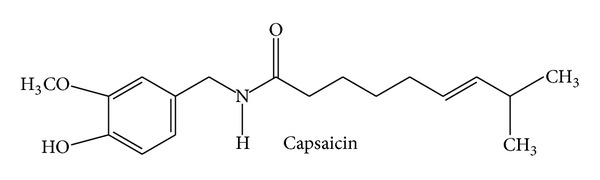


Capsaicin (Figure 4), (E)-N-[(4-hydroxy-3-methoxyphenyl)methyl]-8-methylnon-6-enamide, is a very hydrophobic alkaloid produced by chili peppers (*Capsicum* species; Solanaceae) and is responsible for the typical pungency/spiciness of the fruits of the genus* Capsicum*. It has traditionally been used as a topical rubefacient and counterirritant to relieve pain of muscles and joints. A capsaicin 8% cutaneous patch has recently been approved by the authorities in the EU for the use against neuropathic pain in nondiabetic adults and in the US against neuropathic pain associated with postherpetic neuralgia. Interestingly, the research on capsaicin led to the discovery of the transient receptor potential channel vanilloid subfamily member 1 (TRPV1), which is the direct target of capsaicin [56]. TRPV1 is a nonselective cation channel with high preference for Ca^2+^ and is mainly located in nociceptive neurons. It is activated by chemical and physical stimuli, such as heat, low pH, capsaicin, and certain inflammatory mediators [57]. Prolonged activation of TRPV1 by capsaicin is discussed to cause desensitization and, thus, reduced pain sensation [58]. Beyond pain, some few studies also found an anti-inflammatory potential of capsaicin: it can inhibit paw inflammation in arthritic rats [59] and ethanol-induced inflammation of the gastric mucosa in rats [60]. Moreover, capsaicin was reported to inhibit COX-2 activity, iNOS expression, and the NF*κ*B pathway in macrophages in a TRPV1 independent way [61]. Regarding a clinical influence on inflammatory conditions, capsaicin was evaluated in a recent systematic review by the Cochrane Collaboration: topical capsaicin was reported not to be effective against osteoarthritis [62]. In contrast, one meta-analysis found enough evidence to conclude that capsaicin is effective in the management of osteoarthritis, although the authors pinpointed that there is a paucity of randomized clinical trials [63]. This is in line with a further meta-analysis reporting that capsaicin alleviates osteoarthritic pain [64]. Regarding future therapeutical enhancements, 24 clinical trials are planned or are currently recruiting, as listed on http://www.clinicaltrials.gov/. In most of these studies capsaicin is used as a model substance to induce pain or as a diagnostic tool (provocation test). Trials that investigate the therapeutical potential of capsaicin analyze its potential as preemptive (prior to surgery) analgesic in patients undergoing amputation of a limb and its action on neuropathic pain from critical ischemia (predominantly in hands and feet) and on chronic pain from artificial arteriovenous fistulae (for hemodialysis) in patients with end-stage renal failure. Moreover, capsaicin will be tested against persistent pain after inguinal herniotomy and against the impaired swallow response in stroke patients with oropharyngeal dysphagia. Another study will evaluate the mechanism behind the action of capsaicin against idiopathic rhinitis. No trial deals with anti-inflammatory effects of capsaicin. In summary, in contrast to neuropathic pain, the field of capsaicin and inflammation is not very advanced and will stay on that level in the near future due to a lack of clinical studies.

### 2.5. Epigallocatechin-3-gallate

**Figure 5:**
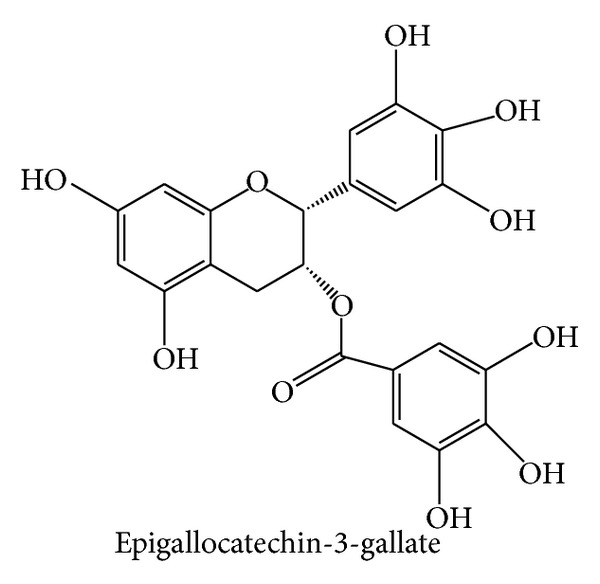


Epigallocatechin-3-gallate (Figure 5), [(2R,3R)-5,7-dihydroxy-2-(3,4,5-trihydroxyphenyl)-3,4-dihydro-2H-chromen-3-yl]3,4,5-trihydroxybenzoate, commonly abbreviated as EGCG, is an ingredient of green tea,* Camellia sinensis* (Theaceae). It is the most prominent member of the family of green tea catechins (polyphenols) and accounts for 50–80% of all catechins in a cup of green tea [65]. The number of reports on its biological activity is huge. EGCG was found to exert profound anti-inflammatory, antioxidant, anti-infective, anticancer, antiangiogenetic, and chemopreventive effects [65–69]. Also the knowledge about the cellular and molecular mechanisms is extremely broad: EGCG promotes cell growth arrest and induces apoptosis by affecting regulatory proteins of the cell cycle and inhibition of NF*κ*B [69–71]. Furthermore, it inhibits growth factor-dependent signaling (e.g., of EGF, VEGF, and IGF-I), the MAPK pathway, proteasome-dependent degradation, and expression of COX-2 [72, 73]. Even molecular targets of EGCG have been identified. It seems to directly interact with and to modulate the character of membrane lipid rafts, which explains the ability to alter signaling processes of growth factor receptors [74–76]. Furthermore, EGCG inhibits telomerase, topoisomerase II, and DNA methyltransferase 1, thereby affecting the functions of chromatin [77–79]. Surprisingly, however, despite the promising preclinical findings and the thorough mechanistic insights, clinical studies in the context of inflammation are largely lacking. One small study analyzed the action of green tea and an extract thereof on biomarkers of inflammation (e.g., adiponectin, CRP, IL-6, IL-1*β*, sVCAM-1, and sICAM-1) in obese patients with metabolic syndrome. After 8 weeks of treatment, biomarker levels were not changed by green tea [80]. Another trial reported a beneficial impact of topical EGCG treatment on acne vulgaris, which might be at least in part due to anti-inflammatory effects [81]. Interestingly, in 2006, a green tea extract was approved as a prescription drug for the topical treatment of genital and anal warts (condylomata acuminata). This great advancement fueled further research to expand the indications of EGCG. What will be clinically analyzed in the next years? 17 open studies are listed on http://www.clinicaltrials.gov/. EGCG will be tested for its effects on albuminuria in diabetic nephropathy as well as for its action in patients with cardiac amyloid light-chain amyloidosis, with muscular dystrophy of the Duchenne type, with Alzheimer's disease (early stage), with Down syndrome, with fragile X syndrome, with Huntington's disease, and with multiple-system atrophy. Moreover, trials will analyze the potential of EGCG on reactivation of the Epstein-Barr virus in remission patients and on preventing colon polyps in patients at high risk for recurrent colon adenoma. Further studies will examine whether EGCG affects insulin resistance, whether gargling with EGCG prevents influenza infections in teenagers, and whether topical EGCG exerts an anticarcinogenic potential in patients with superficial basal cell carcinoma. Obviously, the trials will not investigate classic inflammatory disorder, although some of the mentioned diseases are associated with inflammatory processes (e.g., Alzheimer's disease or insulin resistance). Nevertheless, it is very likely that EGCG will experience an expansion of its indication in the future.

### 2.6. Quercetin

**Figure 6:**
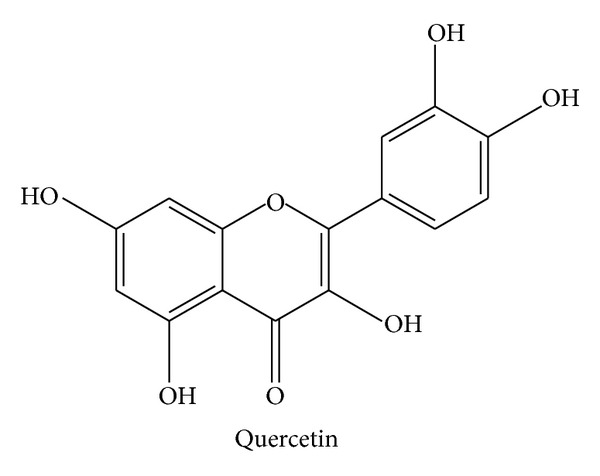


Quercetin (Figure 6), 2-(3,4-dihydroxyphenyl)-3,5,7-trihydroxychromen-4-one, a flavonol, belongs to the class of flavonoids, which is a large family of polyphenols representing very widely spread secondary plant metabolites. Quercetin is found in a great variety of food, such as apples, grapevines, berries, broccoli, red onions, capers, or tea. As with the above discussed compounds, also quercetin exerts a large spectrum of biological effects: anti-inflammatory [82], anti-infectious [83], antioxidant [84], anticancer/chemopreventive [85, 86], neuroprotective [87], antihypertensive [88, 89], and blood glucose-lowering [90] properties have been reported. Also the mechanisms behind these actions are very broad and have been characterized intensively. Quercetin scavenges reactive oxygen and nitrogen species [84], targets prominent proinflammatory signaling pathways, such as STAT1, NF*κ*B, and MAPK [91, 92], and inhibits infectivity of target cells and replication of many types of viruses [93]. Moreover, phosphodiesterases (PDEs) were suggested to be affected by quercetin [94], as well as topoisomerases I and II [95] and Mcl-1 [86]. Most importantly, quercetin was identified as a broad-spectrum kinase inhibitor [96, 97]. Has all this knowledge been translated into therapy or prevention? Several studies on inflammatory parameters in humans have been performed in the last years: one trial evaluated the effect of quercetin on biomarkers of inflammation depending on the apolipoprotein E genotype of healthy men. Although risk factors of cardiovascular disease were improved, quercetin exerted a slight proinflammatory effect (increased levels of TNF*α*) [98]. It was reported that quercetin had no action on the levels of the proinflammatory cytokine IL-6 after repeated sprint exercise [99]. In sarcoidosis patients, quercetin reduced markers of inflammation (TNF*α* and IL-8) [100]. In healthy females, quercetin did not alter blood leukocyte subsets, granulocyte oxidative burst or phagocytosis activity, IL-6, or TNF*α* plasma levels [101]. Of note, no trial reports on the improvement of clinical parameters of inflammatory diseases (severity and incidence). Almost ten clinical studies are registered on http://www.clinicaltrials.gov/ that are going to use pure quercetin as a pharmacological compound. With respect to inflammatory disorders, quercetin will only be analyzed in two-phase 1-2 trials for its safety and dose-response relationship in chronic obstructive pulmonary disease (COPD). In the field of diabetes, quercetin will be tested in a phase 2 study for an effect on blood glucose and blood vessel function in type 2 diabetes. Quercetin will also be given to obese patients (with or without diabetes type 2) to test its action on glucose absorption (glucose tolerance test). Regarding cancer, it will be tested whether quercetin modulates levels of prostate-specific antigen (PSA) and whether it can prevent prostate cancer. Moreover, in a pilot study, quercetin will be used in children suffering from Fanconi anemia (safety and pharmacokinetics). Thus, quercetin will undergo very interesting studies that might lead to a profound advancement of knowledge about its clinical efficacy. However, inflammatory diseases are not the main topic of current research.

## 3. Conclusion

State-of-the-art clinical intervention studies, that is, randomized double-blind placebo-controlled trials, are the gold standard for testing whether a substance has a therapeutical or preventive potential. The field of plant-derived compounds often experiences “hope and hype” phases. Opinions and assumptions about an action in humans are rapidly disseminated once promising* in vitro* and* in vivo* findings have been generated. Unfortunately, the step towards a sound and significant clinical trial is difficult, very laborious, long-ranging, and—most importantly—extremely expensive, which means that it is impossible in many cases. Thus, this research area often suffers from either inadequately performed or a low number of studies. In this minireview, we did try not only to pinpoint deficiencies, but also to highlight positive developments that will hopefully lead to an advancement of prevention or therapy of diseases.

## Abbreviations

AP-1: Activator protein-1
COPD: Chronic obstructive pulmonary disease
COX: Cyclooxygenase
CRP: C-reactive protein
EGCG: Epigallocatechin-3-gallate
EGF: Epidermal growth factor
eNOS: Endothelial nitric oxide synthase
IGF: Insulin-like growth factor
iNOS: Inducible nitric oxide synthase
ICAM: Intercellular adhesion molecule
IL: Interleukin
LOX: Lipoxygenase
MAPK: Mitogen-activated protein kinase
NF*κ*B: Nuclear factor *κ*B
PDE: Phosphodiesterase
PPAR: Peroxisome proliferator-activated receptor
PSA: Prostate-specific antigen
SIRT: Sirtuin
sICAM: Soluble intercellular adhesion molecule
STAT: Signal transducer and activator of transcription
sVCAM: Soluble vascular cell adhesion molecule
TNF: Tumor necrosis factor
TRPV1: Transient receptor potential channel vanilloid subfamily member 1
VEGF: Vascular endothelial growth factor.
